# An assessment of evidence to inform best practice for the communication of acute venous thromboembolism diagnosis: a scoping review

**DOI:** 10.1016/j.rpth.2025.102835

**Published:** 2025-03-23

**Authors:** Samarth Mishra, Frederikus A. Klok, Grégoire Le Gal, Kerstin de Wit, Aviva Schwartz, Dieuwke Luijten, Parham Sadeghipour, Julie Bayley, Scott C. Woller

**Affiliations:** 1 Medical College of Georgia, Augusta, Georgia, USA; 2 Department of Medicine – Thrombosis and Hemostasis, Leiden University Medical Center, Leiden, the Netherlands; 3 Department of Medicine, University of Ottawa, Ottawa, Ontario, Canada; 4 Department of Medicine, Queens University, Kingston, Ontario, Canada; 5 Vasculearn Network, Brookline, Massachusetts, USA; 6 Vascular Disease and Thrombosis Research Center, Rajaie Cardiovascular Institute, Tehran, Iran; 7 Northeastern University London (previously University of Lincoln UK), London, United Kingdom; 8 Department of Medicine, University of Utah School of Medicine, Salt Lake City, Utah, USA

**Keywords:** communication, deep vein thrombosis, pulmonary embolism, venous thromboembolism, physician-patient relations

## Abstract

**Background:**

Physician communication with patients is a key aspect of excellent care. Scant evidence exists to inform best practice for physician communication in patients diagnosed with pulmonary embolism and deep vein thrombosis, collectively referred to as venous thromboembolism (VTE).

**Objectives:**

The aim of this study was to summarize the existing literature on best practices for communication between healthcare providers and patients newly diagnosed with VTE.

**Methods:**

We performed a scoping review to report existing literature on best practices for physician-patient communication and the diagnosis and management of acute VTE. Manuscripts on communication between healthcare professionals and patients presenting with acute VTE and acute vascular disease presentations that included atrial fibrillation and acute coronary syndrome were identified. Two authors independently reviewed studies for eligibility and a consensus determined article inclusion. The manuscripts were further categorized into 2 categories: best practices in communication and unmet needs in communication. Data aggregation was achieved by a modified thematic synthesis.

**Results:**

Among 345 initial publications, 22 manuscripts met inclusion criteria, with 11 addressing VTE, 5 pulmonary embolism, 4 deep vein thrombosis, 1 atrial fibrillation, and 1 acute coronary syndrome. Eleven manuscripts addressed communication of VTE diagnosis, while 12 focused on communication of VTE treatment. Eleven manuscripts identified unmet communication needs, and 14 addressed best practices. Our review showed that good communication enhanced satisfaction, while suboptimal communication was associated with emotional, cognitive, behavioral, social, and health systems adverse effects.

**Conclusion:**

Scant literature guides best practices for communicating VTE diagnosis and treatment. Further research is necessary to establish practices for improving communication with VTE patients.

## Essentials

• Excellent physician-patient communication is a key aspect of quality care.
• Limited evidence informs physician-patient communication in acute venous thromboembolism.
• We summarize existing evidence for physician-patient communication in venous thromboembolism.
• Further research is needed to improve communication with venous thromboembolism patients.

## 1 Introduction

Venous thromboembolism (VTE) includes pulmonary embolism (PE) and deep vein thrombosis (DVT). PE is the third leading cause of cardiovascular death and the number one cause of preventable hospital-associated mortality [1,2]. Increasingly, VTE is being diagnosed and treated during a brief emergency department (ED) encounter, which limits the time that healthcare providers can provide patient education and respond to patients’ questions [3]. Physician-patient communication is a key influencer of patients’ perception of their current and future health state [4].

The physician-patient relationship is foundational for quality medical care. Effective physician-patient communication helps patients feel safe in stressful situations and instills confidence in the provider. However, VTE is frequently diagnosed in the ED, which is a chaotic environment with brief encounters that are often stressful. Furthermore, delivering the diagnosis of VTE is a form of breaking bad news, a known inhibitor to patient comprehension, and the capacity to internalize and retain information. For other conditions such as a cancer diagnosis, significant evidence exists that physician-patient communication affects provider trust and influences patient anxiety [5]. In the diagnosis of chronic diseases, such as diabetes, high-quality physician-patient communication is associated with better patient well-being and self-care [5]. Comparatively, less is known about physician-patient communication, patient recovery, and well-being following a VTE diagnosis. Key aspects of decision-making in thrombosis medicine include the discussion surrounding the balanced risk vs the benefit of invasive procedures, usually in the immediate term of diagnosis, and the duration of anticoagulation therapy once the VTE treatment phase is complete.

Our study had 2 aims. First, to summarize the existing literature on best practices for physician-patient communication surrounding a VTE diagnosis, and second, to identify knowledge gaps that would inform future work to optimize communication surrounding acute VTE diagnosis.

## 2 Methods

### 2.1 Scoping literature review

PubMed, CINAHL, Web of Science, and the Cochrane Library Databases were searched for medical subject headings terms, title/abstract terms, and key categories that addressed physician-patient communication and VTE from database inception through June 21, 2023 (original search, Figure). Due to the paucity of relevant articles identified, the search was then expanded to include vascular conditions in addition to VTE, including conditions such as atrial fibrillation and acute coronary syndrome (ACS; enhanced search; Figure). The search criteria are presented in Table 1, and the search protocol was adherent with the Preferred Reporting Items for Systematic reviews and Meta-Analyses extension for scoping reviews methodology [6].

**Figure:**
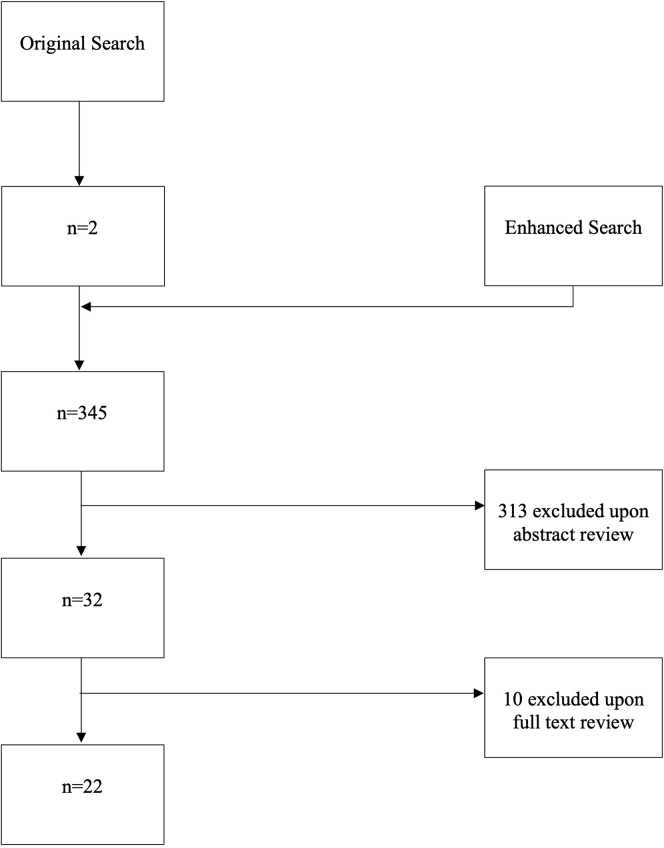
Consolidated standards of reporting trials diagram for search methodology.

**Table 1:** Search strategy with medical subject headings (MeSH) terms and category titles.

Database	Search criteria 1	Search criteria 2	Search criteria 3
PubMed	“Venous Thrombosis” (MeSH) OR “Pulmonary Embolism” (MeSH) OR “DVT” OR “VTE” OR “PE” OR “Deep Venous Thromboses” OR “Deep Venous Thrombosis” OR “Venous Thromboembolism” (MeSH)	“Physician-Patient Relations” (MeSH) OR “Nonverbal Communication” (MeSH) OR “Interpersonal Relations” (MeSH) OR “Communication” (MeSH) OR “Patient Participation”(MeSH) OR “Social Participation” (MeSH) OR “Psychology” (MeSH)	“Vascular Diseases” (MeSH)
CINAHL	“MH Venous Thrombosis +”	“Physician-patient relations” OR (“Psychology+”) OR “Engagement”	“Vascular Diseases +”
Web of Science	“Venous Thrombosis”	“Physician-Patient Relations”	“Vascular Diseases”
Cochrane Library	"Venous Thrombosis" (MeSH) OR “Venous Thromboembolism” (MeSH)	“Physician-Patient Relations” (MeSH) OR “Psychology” (MeSH)	“Vascular Diseases” (MeSH)

DVT, deep vein thrombosis; PE, pulmonary embolism; VTE, venous thromboembolism.

Because in scoping reviews, a search of the gray literature may be considered, out of an abundance of caution, we performed a Google Scholar search and characterized the first 20 results as being relevant to our research question or not, and whether the results added novel information or not. Eighteen of the 20 results were not relevant to the research question. One of the 20 results was identified in our search [7], and the other [8] did not include information that was additively novel to the information identified by the *a priori* search criteria.

### 2.2 Eligibility criteria

Full-text manuscripts written in English, Spanish, German, Dutch, and French that addressed the topic of healthcare provider communication with patients experiencing acute VTE or vascular conditions were eligible for inclusion.

### 2.3 Study selection

Two authors (S.M. and F.A.K.) independently reviewed eligible titles and abstracts. Disagreements were resolved by discussion, and in the case of persistent disagreement, a third author (S.C.W.) was available to adjudicate any disagreement by a simple majority.

### 2.4 Data extraction and synthesis

Data aggregation was achieved by a modified thematic synthesis [9]. Thematic synthesis is a method of data extraction that permits aggregations of themes and has been described in studies that address questions of patient perspective and experience [10]. Our method differed from that formerly described by Thomas and Harden [9], as we adopted a synthesis of the principal theme of the manuscript, as opposed to a “line-by-line” coding that was out of scope for our review.

Two authors (S.M. and S.C.W.) independently reviewed each manuscript and labeled each based on thematic focus.

Manuscripts were categorized into areas of clinical focus that included VTE, PE, and DVT, and the results of the “vascular disease” search, including ACS and atrial fibrillation. Articles were also grouped based on whether the manuscript addressed diagnosis or treatment, and finally, articles were grouped based on which aspect of communication (identification of unmet needs or provision of best practice) each addressed.

## 3 Results

Following the initial search that yielded 2 citations, the final systematic search identified 345 manuscripts. A total of 32 manuscripts were identified for full-text analysis, and 22 manuscripts met the inclusion criteria (Figure) [7,[11], [12], [13], [14], [15], [16], [17], [18], [19], [20], [21], [22], [23], [24], [25], [26], [27], [28], [29], [30], [31], [32], [33], [34], [35], [36], [37], [38], [39], [40], [41]].

An initial assessment of the articles led to the creation of 4 categories for descriptive purposes, presented in Table 2. The primary focuses of the included manuscripts were VTE (11), PE (5), DVT (4), atrial fibrillation (1), and ACS (1). Eleven manuscripts addressed communication about diagnosis, and 12 manuscripts addressed communication about treatment. Eleven manuscripts provided information surrounding the identification of unmet needs in communication, and 14 provided guidance regarding best practices (Table 2).

**Table 2:** Categorization of included manuscripts.

Manuscript descriptive categories	Primary focus: VTE	Primary focus: PE	Primary focus: DVT	Primary focus: AF	Primary focus: ACS
Communication in diagnosis	[7,12,15,17,28]	[16,32,33,37]	[36]		[14]
Communication in treatment	[17,19,20,25,28,40]	[16,26,37]	[18,30]	[23]	
Unmet needs in communication (Table 3)	[7,12,15,17, 20,25,28,40]	[16,37]	[24]		
Best practice in communication (Table 4)	[7,19,20,34,40]	[16,26,32,33]	[18,30,36]	[23]	[14]

ACS, acute coronary syndrome; AF, atrial fibrillation; DVT, deep vein thrombosis; PE, pulmonary embolism; VTE, venous thromboembolism.

### 3.1 Impact of physician-patient communication in VTE

Our review identified several areas of patient well-being influenced by physician-patient communication. de Wit et al. [12] described that poor communication resulted in patient anxiety, and Etchegary et al. [15] enumerated the “Zones of Relevance” that physician communication impacts, including emotional, cognitive, behavioral, social, and health services. Furthermore, Genge et al. [17] identified limitations of clinical trial design in the VTE/vascular space and stated that the physical, emotional, and psychiatric impacts of VTE are beyond the objective outcomes typically measured in clinical trials. However, existing evidence suggests the importance of VTE diagnosis and treatment communication on patient psychological health. In an interview study, 2 of 72 patients diagnosed with acute PE experienced “ongoing and untreated psychological distress” that met the criteria for post-traumatic stress disorder (PTSD) [37]. Patients who experienced ongoing psychological distress tended to recall poor communication at the time of diagnosis [37].

### 3.2 Influence of health literacy on communication effectiveness in VTE

Several manuscripts point to patient health literacy and patient resource readability as central aspects of VTE communication. Fischer et al. [16] proposed a role for questionnaires to “identify patients with low health literacy who may require additional support from the healthcare system…” San Norberto et al. [28] identified that “venous thrombosis patient education materials produced by leading medical societies have readability scores that are above the recommended levels.” The pivotal role of health literacy in patient satisfaction was described in a study of 2154 VTE patients receiving oral anticoagulation treatment where “limited [health literacy] was associated with lower [oral anticoagulation] treatment satisfaction…” [25]. Efforts in making VTE communication easily comprehendible and transmissible to the general population have shown success. Easy-to-understand communication to heighten public awareness of VTE through DVT public awareness campaigns has been found to increase DVT diagnosis rates [36].

### 3.3 Timing and completeness of VTE communication

Evidence exists that physician communication at the time of the VTE diagnosis falls short of patient expectations. A study of telephone interviews with patients prescribed compression stockings (*n* = 12) revealed that patients received little to no information regarding their compression stockings [24]. Likewise, a French study that included 103 VTE patients revealed that “more than 75% of patients reported that no physician warned them about risks of anticoagulation, long-term complications of venous thromboembolic disease or its prevention” [20].

### 3.4 The importance of individualized approaches

A key aspect of effective communication in the acute diagnosis and management of VTE and vascular disease is understanding individual patient values and preferences. However, as evidenced in a systematic review of 48 studies conducted as part of a guideline review [23], patient values and preferences toward treatments can vary, which then requires more investment to understand values in context. One study that focused on patient satisfaction with PE testing in the ED [33] found that patient satisfaction arose from addressing the patient’s primary concern, providing individualized care, utilizing imaging for diagnosis (eg, computed tomography scans), and the perceived confidence exuded by the treating physician [33]. A separate study of patient International Normalized Ratio self-testing found that patient engagement was more likely with personalized physician-patient communication [19].

### 3.5 The need for accurate disclosure and actively addressing difficult discussions

The communication of an acute VTE diagnosis involves ethical variables, especially when decision-making may significantly impact a patient’s future quality of life. Physician-patient collaboration in medical decision-making is an important satisfier when stark differences in possible outcomes exist [18]. A study centered on the discussion of thrombolysis in the event of a submassive PE highlighted that engaging in difficult conversations engenders patient trust and satisfaction [26]. Difficult conversations include those that communicate medical errors and unexpected, “incidental” findings. A study of 971 patients that assessed patient satisfaction found that perceived mistakes in medical care were strongly associated with dissatisfaction, and the authors suggested that interventions aimed at the reduction, acknowledgment, and communication of errors might improve patient satisfaction [40]. Similarly, in a study assessing the communication of incidental findings, Sonis et al. [32] stated that “communication of clinically significant incidental findings identified in the ED to patients and their primary care physicians is essential” for patient satisfaction.

### 3.6 Specialized but not role-restricted communication

Evidence exists that the best practice for communication in the domain of acute VTE is a “team sport.” A Turkish study that included nurse-led DVT training on inpatient protective self-care practices found that nurse education improved patient comprehension [30]. Facilitating specialists in thrombosis endorsed that “information provided by vascular medicine physicians was clearer and more complete” when compared with that of a nonspecialist physician [20].

### 3.7 Awareness of physician communication style and the medicalization of information

Finally, best practice includes that the physician be mindful of overt (ie, patient health literacy or language fluency) aspects of communication and indirect factors of communication (eg, cultural sensitivities and body language). Hernandez-Nino et al. [7] identified that aspects of a healthcare encounter may inadvertently incite fear or reassurance. A physician’s word choice (use of medical jargon), behavior, balance of fear vs reassurance, and the amount of information provided are impactful [7]. Edelman et al. [14] evaluated the impact of native language on encounters in the acute-care setting. He stated, “…native language concordance acts as a protective factor for patient-clinician interpersonal care in the acute setting, regardless of native language or English proficiency” [14]. Literature has shown that it is possible to provide information to patients that they are able to comprehend rather easily. Takara et al. [34] created a VTE information manual for the lay population, which 97.5% of readers deemed as easily understandable, and 90.0% of readers found the material of interest.

Manuscripts that discussed unmet needs in communication are referenced in Table 3, and manuscripts that discussed best communication practices are referenced in Table 4.

**Table 3:** Manuscripts detailing unmet needs in communication.

Article title	Author(s)	Country	Year	Study/article type	Summative statement
“What information patients require on graduated compression stockings.” [24]	May et al.	England	2006	Qualitative – patient interviews/surveys	*N* = 12 patients in the study indicated that those who were using compression stockings had little or no information about their stockings.
“Patient Satisfaction With Venous Thromboembolism Treatment” [40]	Webb et al.	USA	2019	Qualitative – patient interviews/surveys	Perceived mistakes during care for VTE was strongly associated with VTE care dissatisfaction.
“Roles of the general practitioner and the vascular medicine physician for patient education concerning venous thromboembolism: The patient’s perspective.” [20]	Le Collen et al.	France	2019	Qualitative – patient interviews/surveys	Calls for standardization of VTE patient education and communication of essential educational messages pertaining to VTE.
“Communication at diagnosis of venous thromboembolism: Lasting impact of verbal and nonverbal provider communication on patients.” [7]	Hernandez-Nino et al.	USA	2022	Qualitative – patient interviews/surveys	Indicates that nonverbal communication and utilization of medical jargon can influence a patient’s response and can, in some cases, have a negative impact on patient-provider communication.
“The psychological impact of pulmonary embolism: a mixed-methods study” [37]	Tran et al.	Canada	2021	Mixed methods	Two of 72 patients were found to meet the diagnosis of PTSD due to ongoing psychological distress following a PE diagnosis.
“Health literacy in patients with pulmonary embolism: development and validation of the HeLP (Health Literacy in Pulmonary Embolism) – Questionnaire.” [16]	Fischer et al.	Germany	2023	Mixed methods	Proposed the development of an assessment tool to evaluate patient health literacy and which patients may require additional healthcare support.
“Psychosocial aspects of venous thromboembolic disease: an exploratory study.” [15]	Etchegary et al.	Canada	2008	Exploratory	VTE diagnosis communication can impact “Zones of Relevance,” which include emotional, cognitive, behavioral, social, and health services. Patient knowledge deficits in the VTE setting raise questions about current knowledge provisions and consent protocols.
“Do physicians contribute to psychological distress after venous thrombosis?” [12]	De Wit	Canada	2022	Commentary	A mismatch between physician goals and patient needs in the VTE setting is contributing to increased prevalence of patient anxiety among patients with VTE. The mental recovery of patients can be improved with thought, empathy, and good communication skills.
“Evaluation of patients’ experience and related qualitative outcomes in venous thromboembolism: a scoping review” [17]	Genge et al.	Canada	2022	Scoping review	Clinical trials studying VTE often are unable to measure the physical, emotional, and psychiatric components that come alongside a VTE diagnosis.
“Health Literacy and Treatment Satisfaction Among Patients with Venous Thromboembolism” [25]	Mefford et al.	USA	2022	Retrospective cohort	Patients with limited health literacy were found to have lower oral anticoagulant treatment satisfaction.
“Readability of patient educational materials in venous thrombosis: analysis of the 2021 ESVS guidelines and comparison with other medical societies information.” [28]	San Norberto et al.	Spain	2022	Cross-sectional	Readability of VTE patient education materials by leading societies were found to have readability scores above the recommended levels.

ESVS, European Society for Vascular Surgery; PE, pulmonary embolism; PTSD, post-traumatic stress disorder; VTE, venous thromboembolism.

**Table 4:** Manuscripts detailing best communication practices.

Article title	Author(s)	Country	Year	Study type	Summative statement
“Technology-assisted self-testing and management of oral anticoagulation therapy: a qualitative patient-focused study.” [19]	Kuljis et al.	UK	2016	Qualitative – patient interviews/surveys	Patient engagement and involvement are likely to be realized more when patients receive timely, individualized, and face-to-face care.
“Patient Satisfaction With Venous Thromboembolism Treatment” [40]	Webb et al.	USA	2019	Qualitative – patient interviews/surveys	Mentions importance in disclosing any reasons for treatment mistakes, diagnostic mistakes, and/or delayed treatment to preserve a healthy physician-patient relationship.
“Roles of the general practitioner and the vascular medicine physician for patient education concerning venous thromboembolism: The patient’s perspective.” [20]	Le Collen et al.	France	2019	Qualitative – patient interviews/surveys	With respect to VTE diagnosis communication, patients reported having better communication with vascular-specialized physicians compared with general physicians. Calls for a standardized approach for VTE communication.
“Patient values and preferences in pulmonary embolism testing in the emergency department” [33]	Swarup et al.	Canada	2021	Qualitative – patient interviews/surveys	Patients being evaluated for PE in the ED were found to prefer care that was individualized, centered on their primary concern, and care that involved the utilization of medical imaging.
“Communication at diagnosis of venous thromboembolism: Lasting impact of verbal and nonverbal provider communication on patients.” [7]	Hernandez-Nino et al.	USA	2022	Qualitative – patient interviews/surveys	The utilization of simple terminology and single components is likely to improve VTE communication in the acute-care setting. Nonverbal communication and expressions should be an additional area of focus.
“Incorporating patients’ preferences into medical decisions.” [18]	Kassirer	USA	1994	Commentary	Patients should play an active role in medical decision-making, especially when decisions may have differences in possible outcomes.
“Communication of imaging recommendations to ED patients: Pulmonary embolus CT.” [32]	Sonis et al.	USA	2016	Commentary	Communication of incidental findings is essential to pave a pathway for patients to trust their physicians and follow-up/attain further care.
“Patient values and preferences in decision making for antithrombotic therapy: a systematic review” [23]	MacLean et al.	Canada	2012	Systematic review	Patient values and preferences with respect to treatment vary due to prior experiences or health outcomes. Patient preferences should be considered prior to initiating any treatments.
“Effect of a public awareness campaign on the incidence of symptomatic objectively confirmed deep vein thrombosis: a controlled study.” [36]	Tomkowski et al.	USA	2012	Observational – case-control study	Raising public awareness for DVT resulted in increased diagnoses. When the awareness campaign was halted, diagnosis rates dropped to baseline.
“Controversy and consent: achieving patient autonomy in thrombolysis for acute submassive pulmonary embolism.” [26]	Pywell et al.	UK	2015	Case report	Refers to the presentation of a patient with PE and discussion of the difficulties associated with obtaining consent. Ultimately, when a patient and physician have a disagreement over treatment options, it is crucial to involve the patient further in the final decision.
“Nurse-led patient training improves deep vein thrombosis knowledge and self-care practices.” [30]	Serpici and Gursoy	Turkey	2018	Quasi-experimental	Vital to incorporate all members of the medical team together when treating a patient. The study found that when patients were educated and trained by nurses, they had more knowledge and took more responsibility for their own care.
“Development and validation of an informative manual on venous thromboembolism for the lay population” [34]	Takara et al.	Brazil	2020	Methodological study	Development of an informative manual on VTE was found to be understood by 97.5% of readers, with another 90.0% stating the reading was not tiresome.
“Impact of Native Language, English Proficiency, and Language Concordance on Interpersonal Care During Evaluation of Acute Coronary Syndrome.” [14]	Edelman et al.	USA	2022	Prospective cohort	Language concordance serves as a protective factor for the patient-clinician relationship in the acute-care setting.
“Health literacy in patients with pulmonary embolism: development and validation of the HeLP (Health Literacy in Pulmonary Embolism) – Questionnaire.” [16]	Fischer et al.	Germany	2023	Mixed methods	Vital to consider patient health literacy when providing care to patients with VTE.

DVT, deep vein thrombosis; ED, emergency department; PE, pulmonary embolism; VTE, venous thromboembolism.

## 4 Discussion

Patient comprehension, satisfaction, and experience with a VTE diagnosis are linked to communication. We found that the literature that informs best practice in physician-patient communication surrounding acute VTE diagnosis and treatment is limited. To the best of our knowledge, this is the first comprehensive review summarizing existing literature to inform best practices in acute VTE diagnosis and treatment.

Work continues to refine the optimal definition of “best practice” in clinical care. One definition summarizes best practice as “the ‘best way’ to identify, collect, evaluate, disseminate, and implement information about as well as to monitor the outcomes of health care interventions for patients/population groups and defined indications or conditions” [42]. For the purposes of our manuscript, we used best practice as a reliable, replicable pathway to communicate important patient information that advances their well-being.

These findings inform future clinical and research efforts in physician-patient communication; however, they should not be considered exclusive to physician-patient communication but should be applied variably to improve communication among all members of the patient care team. We report evidence that quality communication surrounding the diagnosis and treatment of acute VTE can enhance patient satisfaction and engagement, while suboptimal communication can imbue emotional, cognitive, behavioral, social, and health systems-related adverse effects. Patient health literacy and understanding patient preferences are cornerstones of effective communication. This study serves as the foundation to determine what is known about acute VTE diagnosis and treatment communication and what is presently described as best practice in this area. This information may inform processes to improve communication in acute-care domains. Preexisting literature on effective physician-patient communication for other conditions, such as cancer and diabetes, reveals the vital role that effective communication plays in patient outcomes and experiences. For example, in the delivery of cancer diagnosis communication, it has been found that patients experience far less anxiety and more trust in their physician when the physician communicates in a manner that incorporates empathy and the patient’s prior experiences [5]. Guidance surrounding best communication practices from other conditions may inform the creation of appropriate VTE communication guidelines; however, specific directions, including a “physician best-practice toolkit in VTE communication,” are yet to be established. Additional research is needed to inform best practices in VTE diagnosis/treatment communication.

Our results are inherently limited by the paucity of literature that informs best practices in communication surrounding VTE diagnosis and treatment, and these results were obtained using search terms across 4 databases. We acknowledge that our searched databases may not include the complete body of literature surrounding what is known about physician-patient education and communication. Despite the expansion of the search criteria to other vascular conditions, few (22) articles met the inclusion criteria for this study. Furthermore, while our work highlights the importance of cultural sensitivities in communication, our results are limited because the studies that met the inclusion criteria did not reliably include demographics on patient socio-cultural determinants of health, and therefore, we were not able to analyze the outcomes stratified by racial group or ethnicity. Though our data may be limited in providing specific communication practices for a VTE diagnosis, our study proves useful in identifying unmet needs in VTE diagnosis and treatment.

## 5 Conclusion

We conducted a scoping review of the extant literature regarding communication by the physician to the patient on the acute diagnosis of VTE. The opportunity exists to develop best practice guidance, though further research is needed to inform guidance development and evaluate effectiveness. Understanding the existing guidance and knowledge gaps can serve as a roadmap to create a physician VTE communication toolkit that can provide an evidence-based resource to optimize communication in the acute diagnosis and treatment of VTE.
